# Mechanosensitive expression of the mesenchymal subtype marker connective tissue growth factor in glioblastoma

**DOI:** 10.1038/s41598-022-19175-8

**Published:** 2022-09-02

**Authors:** Thomas James Grundy, Louise Orcheston-Findlay, Eshana de Silva, Thuvarahan Jegathees, Victoria Prior, Farhana Amy Sarker, Geraldine Margaret O’Neill

**Affiliations:** 1 Children’s Cancer Research Unit, Kids Research Institute at the Children’s Hospital at Westmead, Westmead, NSW 2145 Australia; 2 Children’s Hospital Westmead Clinical School, Faculty of Medicine and Health, University of Sydney, Sydney, 2006 Australia

**Keywords:** Cancer, Cancer microenvironment, Mechanotransduction

## Abstract

Mechanical forces created by the extracellular environment regulate biochemical signals that modulate the inter-related cellular phenotypes of morphology, proliferation, and migration. A stiff microenvironment induces glioblastoma (GBM) cells to develop prominent actin stress fibres, take on a spread morphology and adopt trapezoid shapes, when cultured in 2D, which are phenotypes characteristic of a mesenchymal cell program. The mesenchymal subtype is the most aggressive among the molecular GBM subtypes. Recurrent GBM have been reported to transition to mesenchymal. We therefore sought to test the hypothesis that stiffer microenvironments—such as those found in different brain anatomical structures and induced following treatment—contribute to the expression of markers characterising the mesenchymal subtype. We cultured primary patient-derived cell lines that reflect the three common GBM subtypes (mesenchymal, proneural and classical) on polyacrylamide (PA) hydrogels with controlled stiffnesses spanning the healthy and pathological tissue range. We then assessed the canonical mesenchymal markers Connective Tissue Growth Factor (CTGF) and yes-associated protein (YAP)/transcriptional co-activator with PDZ-binding motif (TAZ) expression, via immunofluorescence. Replating techniques and drug-mediated manipulation of the actin cytoskeleton were utilised to ascertain the response of the cells to differing mechanical environments. We demonstrate that CTGF is induced rapidly following adhesion to a rigid substrate and is independent of actin filament formation. Collectively, our data suggest that microenvironmental rigidity can stimulate expression of mesenchymal-associated molecules in GBM.

## Introduction

GBM is the highest-grade brain cancer, characterised by diffuse, infiltrative dissemination into the brain parenchyma^1^. Integrated genomic, transcriptomic, and epigenetic studies have identified molecular GBM subtypes that resemble distinct stages in neurogenesis^2^, recently refined to three predominant subtypes, namely proneural, classical and mesenchymal^3^. Some studies have suggested that the subtypes are associated with differential treatment responses^4^, with the mesenchymal subtype being the most aggressive and carrying the worst prognosis^5^. Studies suggest that tumour plasticity may result in proneural tumours transitioning to a mesenchymal subtype following treatment and relapse^5–7^. This mirrors the progression to treatment resistance that accompanies epithelial to mesenchymal transition in other cancer types^8^. While the incidence of a transition from proneural to mesenchymal in GBM has not been replicated in all studies^3^, a definitive answer is complicated by limited availability of matched tumour samples at pre-treatment and post-recurrence. Nonetheless, understanding the inputs controlling the expression of mesenchymal-associated proteins is an important goal for identifying approaches to improve survival for patients diagnosed with this cancer.

The brain is one of the body’s softest tissues^9^ with values of elastic modulus *E* (describing resistance to elastic deformation) quoted ranging from 0.1 to 13.5 kPa depending on the measurement approach taken^10–14^. There is some controversy around the stiffness of brain malignancies, however; some report that GBM tumours are softer than the surrounding brain^15,16^, whereas others suggest they are stiffer^17^. Further, some studies suggest that GBM may become more rigid after treatment^18^.

The importance of mechanosignals derived from a tumour-secreted rigid, collagen-rich matrix is increasingly appreciated^19^. Cells chiefly sense the mechanical properties of the surrounding environment (mechanosensing) through transmembrane integrin receptors located on the cell surface^20^, which engage the extracellular matrix (ECM), then cluster forming focal adhesions and activating an internal signalling cascade. This promotes actin polymerization, where bundles of polymerized actin filaments (stress fibres) link to the integrin cytoplasmic tail. Myosin motor proteins on the stress fibres then exert contractile force through the focal adhesion onto the ECM^21^. Consequently, in a rigid environment, increased internal contraction is generated in response and via this two-way force transmission, cells sense mechanical forces in the underlying tissue. Concomitantly, the outside-in mechanical signal received through the integrins is converted into biochemical signals.

Reports have demonstrated that GBM reacts to cues from a rigid microenvironment by spreading and forming prominent actin stress fibres^22–24^. These phenotypes are reminiscent of mesenchymal transition, suggesting that a rigid tissue environment may contribute to the GBM mesenchymal program. The mesenchymal subtype is characterized by high level expression of connective tissue growth factor (CTGF)^5^. CTGF expression is mechanosensitive^25–28^ and is one of the most upregulated genes in response to mechanical stress applied to fibroblasts^29^. CTGF is a downstream target of well-known mechanotransductive transcriptional co-activators yes-associated protein (YAP) and transcriptional co-activator with PDZ-binding motif (TAZ)^25,30^. As YAP and its paralog TAZ share a distinctive WW domain that binds exclusively to Pro-Pro-X-Tyr binding motifs^31^, and their individual roles in mechanosignalling have yet to be defined, they are often used either in conjunction or interchangeably. Experiments with drugs that variously induce actin polymerisation and depolymerisation, respectively, have led to the suggestion that mechanosensitive CTGF expression is inversely correlated with the ratio of monomer-to-filamentous actin^32^, although there may be cell type specific differences^33^.

The goal of the present study was to assess whether substrate rigidity induces CTGF in GBM, whether there are differences in rigidity-mediated CTGF expression between cell lines representing different GBM subtypes and whether CTGF regulation is inversely related to monomeric actin levels in GBM.

## Results

### CTGF expression is rigidity sensitive

**Figure 1:**
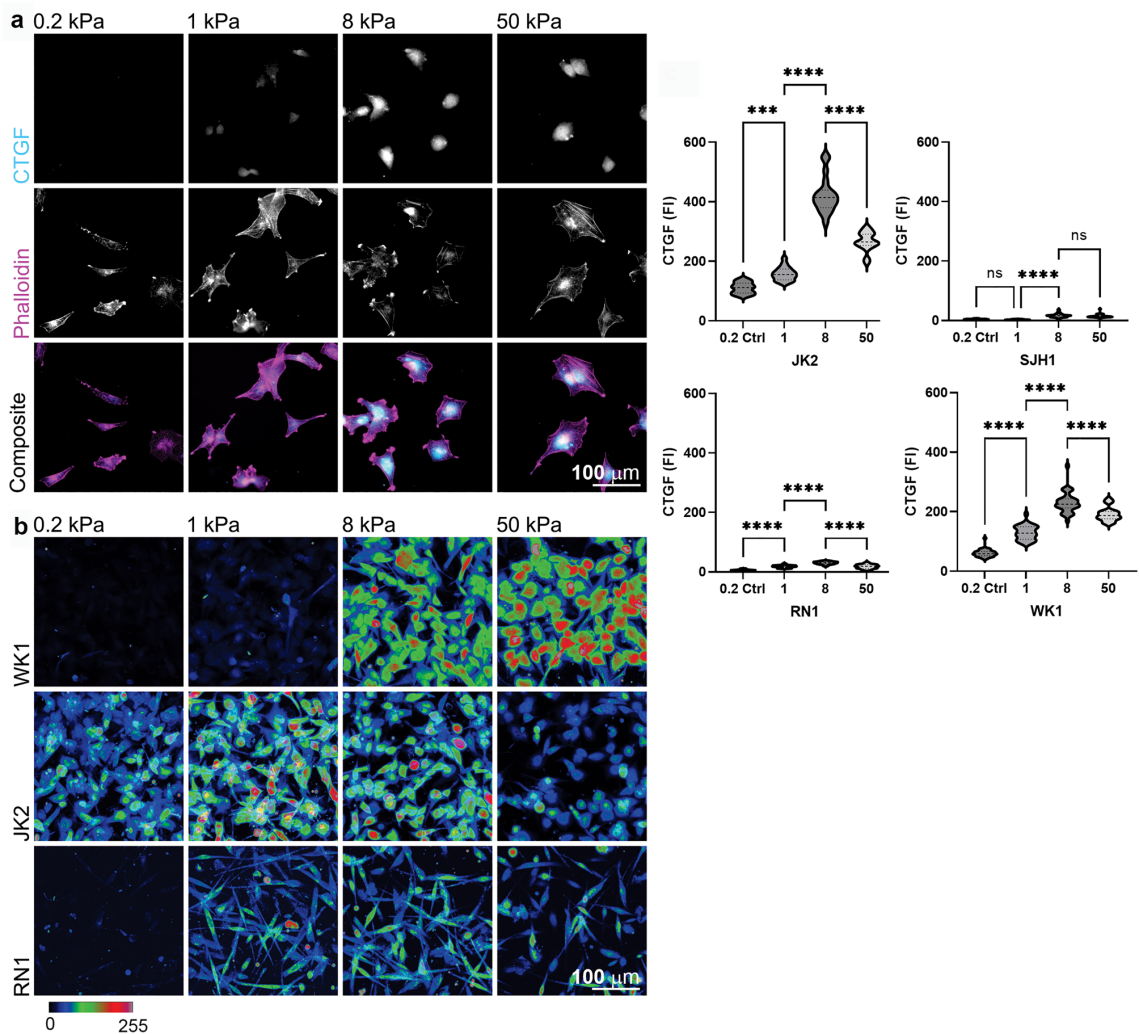
Connective tissue growth factor (CTGF) expression increases with increasing substrate stiffness. (**a**) WK1 cells seeded onto matrix-coupled polyacrylamide (PA) hydrogels (PAGs) of varying stiffness (0.2, 1, 8 or 50 kPa) and immunostained for CTGF. Scale bar 100 µm. (**b**) Colour-coded contrast representations of CTGF immunostaining in WK1, JK2 and RN1 cells grown on matrix-coupled PAGs as indicated. Images show multiple fields of view collapsed into a single image in order to view the CTGF staining across the cell population. Note that the low level of CTGF expression in the SJH1 cells precluded colour-coded representation of this cell line. An example of SJH1 cells immunostained for CTGF expression is instead shown in Supplementary Fig. 2. Image grey values (0–255) represented as varying colour hues as shown. Scale bar 100 µm. (**c**) Graphs show the mean fluorescent intensity of CTGF immunostaining for JK2, SJH1, RN1 and WK1 expressed relative to the cell area. Cells were segmented based upon F-actin counterstaining. Data were pooled from 16 regions of interest per cell line, per condition. Symbols denote statistical significance: ****p < 0.0001; ***p < 0.001; N = not significant (p > 0.05); One-way ANOVA with Tukey’s post-comparison test. Error bars indicate SEM.

Mechanosensitive regulation of the mesenchymal marker CTGF was assessed in primary patient-derived GBM lines grown on Matrigel-coated poly-acrylamide hydrogels (PAGs) in a range of defined stiffnesses emulating brain tissue (0.2, 1 and 8 kPa)^10,11^, and the upper stiffness limit of fibrotic tissue (50 kPa). GBM cell lines WK1 (classical), SJH1 (proneural), RN1 (mesenchymal) and JK2 (mesenchymal) were used to represent each commonly described GBM subtype^34^. CTGF expression was determined by immunofluorescence of fixed cells, allowing individual cells to be analysed to account for any heterogeneity within each population. A representative series of images of CTGF and filamentous-actin staining of WK1 cells is shown (Fig. 1a) and using colour-coded contrast with a rainbow look-up table^35^ to facilitate visualization of differences in CTGF staining (Fig. 1b). Calculation of the mean fluorescent intensity of CTGF per cell suggested that expression peaked in all cell lines on 8 kPa substrates, then decreased on 50 kPa (Fig. 1c). We considered whether the change in CTGF staining intensity might be due to the change in cell spread area in response to the increasing stiffness of the PAGs. To test this we measured cell area and compared this to CTGF intensity. There was no significant correlation between cell area and CTGF intensity (WK1: R2 = 0.778, p = 0.118; JK2: R2 = 0.425, p = 0.348; SJH1 R2 = 0.15, p = 0.612, Pearson’s correlation, two-tailed). Collectively, the data presented suggest mechanosensitive expression of CTGF in all primary GBM lines tested. By contrast, expression of a second mesenchymal marker, CD44, was unaffected by underlying stiffness (Supplementary Fig. 1).

### YAP/TAZ nuclear accumulation is regulated by substrate rigidity

**Figure 2:**
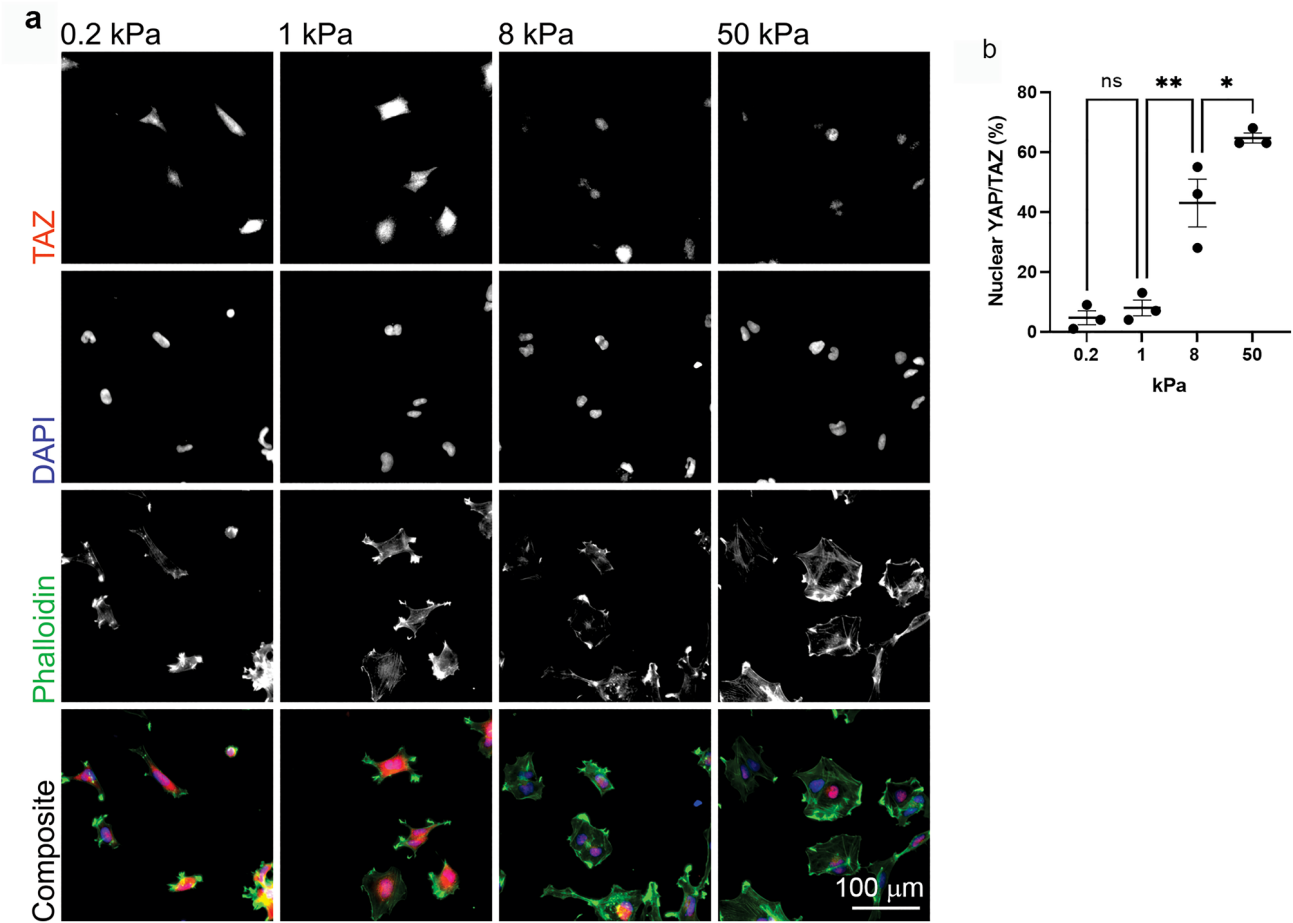
YAP/TAZ nuclear accumulation is regulated by substrate rigidity. (**a**) WK1 cells cultured on matrix-coupled PAGs, immunostained with anti-TAZ antibodies, DAPI to detect nuclei and phalloidin to detect F-actin. (**b**) Quantification of YAP/TAZ nuclear accumulation relative to substrate stiffness. Each data point shows the average score from 3 blinded scorers (details in methods). Symbols denote statistical significance: **** **p < 0.01; *p < 0.05; NS = not significant; One-way ANOVA with Tukey’s post-comparison test. Error bars indicate SEM.

Shuttling of TAZ between the cytoplasm and the nucleus correlates with environment stiffness^25,36^, therefore, TAZ immunostaining was undertaken to assess the activity of mechanosensitive pathways in WK1 cells cultured on 0.2–50 kPa hydrogels. The nuclear/cytoplasmic localisation of TAZ was visualised on each substrate stiffness by immunofluorescence (Fig. 2a) and the percentage of cells with nuclear localisation was then determined (Fig. 2b). The percentage of cells with nuclear YAP/TAZ positively increased with stiffness and was significantly elevated in cells on 8 kPa and 50 kPa gels versus 1 kPa gels. TAZ was > 60% nuclear on 50 kPa gels and < 10% nuclear on 0.2 kPa PAGs. This data indicated that mechanosensitive pathways were active in this primary GBM cell line when cultured as a monolayer on PAGs and reflects previously well-established mechano-dependent regulation of this molecule^25^.

### CTGF expression precedes cell spreading

**Figure 3:**
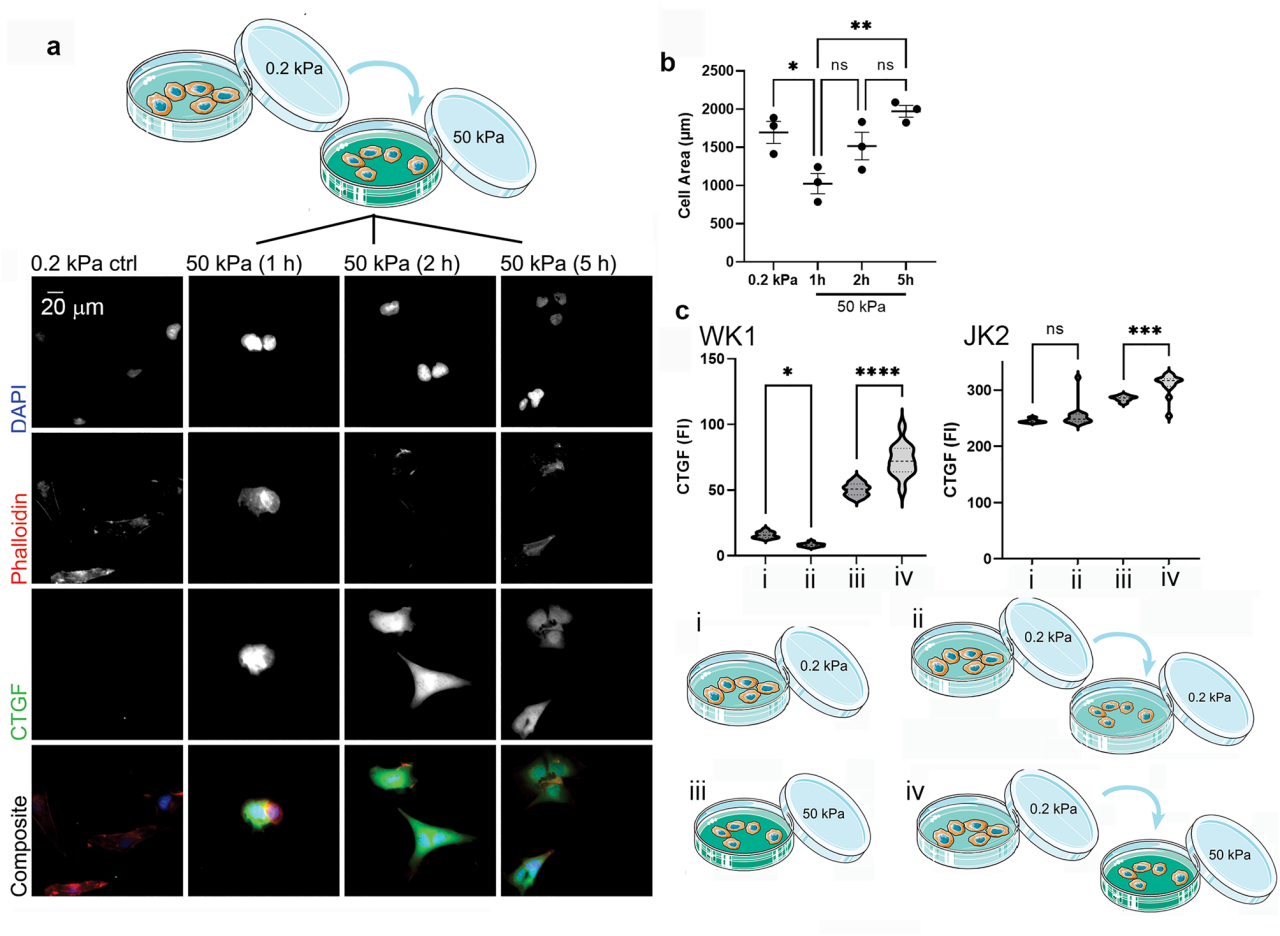
Connective tissue growth factor expression is rapidly induced by a stiff tissue environment. (**a**) WK1 cells grown on 0.2 kPa gels (ctrl) or grown on 0.2 kPa gels, then detached and replated onto 50 kPa gels as per the schematic and for the indicated times. Cells were immunostained with anti-CTGF antibodies, DAPI to detect nuclei and phalloidin to detect F-actin. Scale bar = 20 µm. (**b**) Mean surface area of cells cultured as shown in (**a**). Each data point represents the average from 3 biological repetitions (n = 35 for each respective group). Error bars indicate ± SEM. Symbols denote statistical significance: **p < 0.01; *p < 0.05; NS = not significant (p < 0.05); One-way ANOVA with Tukey’s post-comparison test. (**c**) Plots show fluorescence intensity of CTGF. WK1 and JK2 cells were treated as follows: (i) incubated on 0.2 kPa PAGs, (ii) incubated for 24 h on 0.2 kPa PAGs then transferred to fresh 0.2 kPa PAGs for 1 h, (iii) incubated on 50 kPa PAGs and (iv) incubated for 24 h on 0.2 kPa PAGs then transferred to 50 kPa PAGs for 1 h. Graphs show data pooled from ≥ 13 fields of view. Error bars indicate ± SEM. Symbols denote statistical significance: ****p < 0.0001; ***p < 0.001; *p < 0.05; Ns = not significant (p < 0.05); One-way ANOVA with Tukey’s post-comparison test.

Since mechanosensitive CTGF expression is reported to inversely correlate with the ratio of monomer-to-filamentous actin, CTGF expression was assessed in actively spreading WK1 cells, when actin polymerisation occurs. WK1 cells were first plated onto 0.2 kPa gels overnight to reduce CTGF expression. The following day, cells were detached and replated onto 50 kPa gels (Fig. 3a) and CTGF expression was analysed via immunofluorescence imaging at 1, 2 and 5 h after replating. CTGF levels increased within 1 h of replating on 50 kPa gels (Fig. 3c, WK1 condition (iv)). Notably, 1 h after plating cells onto 50 kPa gels they were significantly smaller than the control cells (Fig. 3b), reflecting a lack of spreading at this early timepoint. Similarly, CTGF levels increased in JK2 cells within 1 h of replating onto 50 kPa gels (Fig. 3c, JK2 condition (iv)). Thus, this confirms upregulation of CTGF prior to cell spreading and suggests that mechano-stimulation of CTGF precedes the polymerisation of the actin cytoskeleton that mediates cell spreading.

Importantly, changes in CTGF expression were not simply a consequence of the replating process. This was demonstrated by analyses of cells that had been first incubated on 0.2 kPa and then been replated directly onto 0.2 kPa PAGs (Fig. 3c(ii)). Under these experimental conditions, CTGF expression in WK1 cells was reduced even further and was unchanged in JK2 cells that had been replated onto 0.2 kPa PAGs. Collectively, these data suggest that increased CTGF is a consequence of cell growth on the rigid 50 kPa substrate environment.

### Mechanosensitive CTGF expression precedes actin filament organization

**Figure 4:**
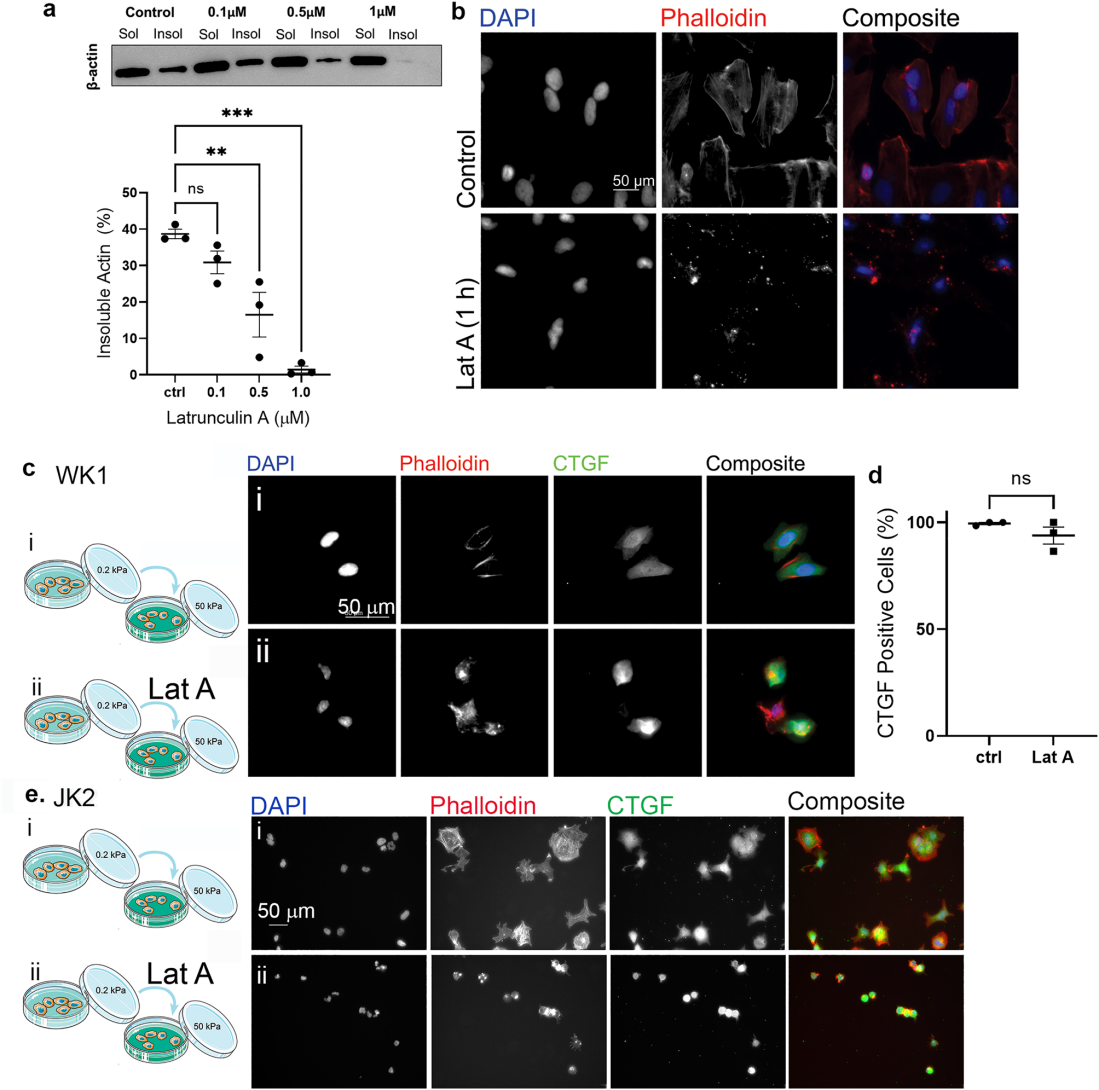
Mechanosensitive CTGF expression precedes actin filament organization. (**a**) The concentration of Latrunculin A required to disassemble actin filaments was determined by western blot analysis of the insoluble versus soluble β-actin fraction following treatment of cells grown in plastic tissue culture dishes with the indicated Latrunculin A concentrations. Data points in graph show mean proportion of insoluble actin in WK1 cells as a percentage of the total β-actin (sum of insoluble plus soluble fraction) from three independent biological repeats. Error bars indicate ± SEM. Symbols denote statistical significance: **p < 0.01; ***p < 0.001; N = not significant (p < 0.05); One-way ANOVA with Tukey’s post-test. (**b**) Control untreated cells and cells treated with 0.5 µM Latrunculin A for 1 h, as indicated, grown on glass coverslips. Cells were fixed and stained with DAPI to show nuclei and phalloidin to detect F-actin. (**c**) WK1 cells cultured on 0.2 kPa gels were detached and replated onto 50 kPa gels either untreated (i, top row, ctrl) or in the presence of 0.5 µM Latrunculin A (ii Lat A, bottom row) as indicated in the schematic. Cells were then fixed and immunostained with anti-CTGF, DAPI to detect cell nuclei and phalloidin to detect F-actin—shown individually in grey scale and as a colour overlay in the final column. (**d**) Quantification of percentage of CTGF positive cells from the conditions described in (**c**). Data points represent three independent biological repeats. Error bars indicate ± SEM. Symbols denote statistical significance: N = not significant (p < 0.05); Students’ *t*-test. (**e**) JK2 cells. Scale bars = 50 µm.

To investigate the specific role of actin stress fibres in this stiffness-mediated effect on CTGF expression, cells were treated with the actin-depolymerising compound Latrunculin A (Lat A). Soluble/insoluble fractions extracted from cells grown on plastic dishes were first visualised and analysed via Western Blot and densitometry, respectively to determine the concentration of Lat A required (Fig. 4a). This revealed a dose dependent increase in the soluble fraction with increasing Lat A concentration, as expected (Fig. 4a), with 0.5 µM Lat A resulting in significantly increased levels of soluble actin. Immunofluorescence of F-actin further confirmed the loss of polymerised actin (actin stress fibres) in cells treated with 0.5 µM Lat A (Fig. 4b).

Having established the conditions of Lat A for effective actin depolymerisation, these conditions were used to directly test whether CTGF expression precedes actin filament organization. WK1 cells were thus seeded onto 0.2 kPa gels overnight and subsequently detached, incubated with 0.5 µM Latrunculin A and replated in the presence of Latrunculin onto 50 kPa gels. Despite Latrunculin-mediated inhibition of stress fibre formation, CTGF expression was induced (Fig. 4cii,d). Similarly, CTGF expression was induced in JK2 cells despite actin disassembly with Latrunculin A prior to replating on 50 kPa gels (Fig. 4eii).

### Mechanosensitive CTGF expression is independent of induced actin organization

**Figure 5:**
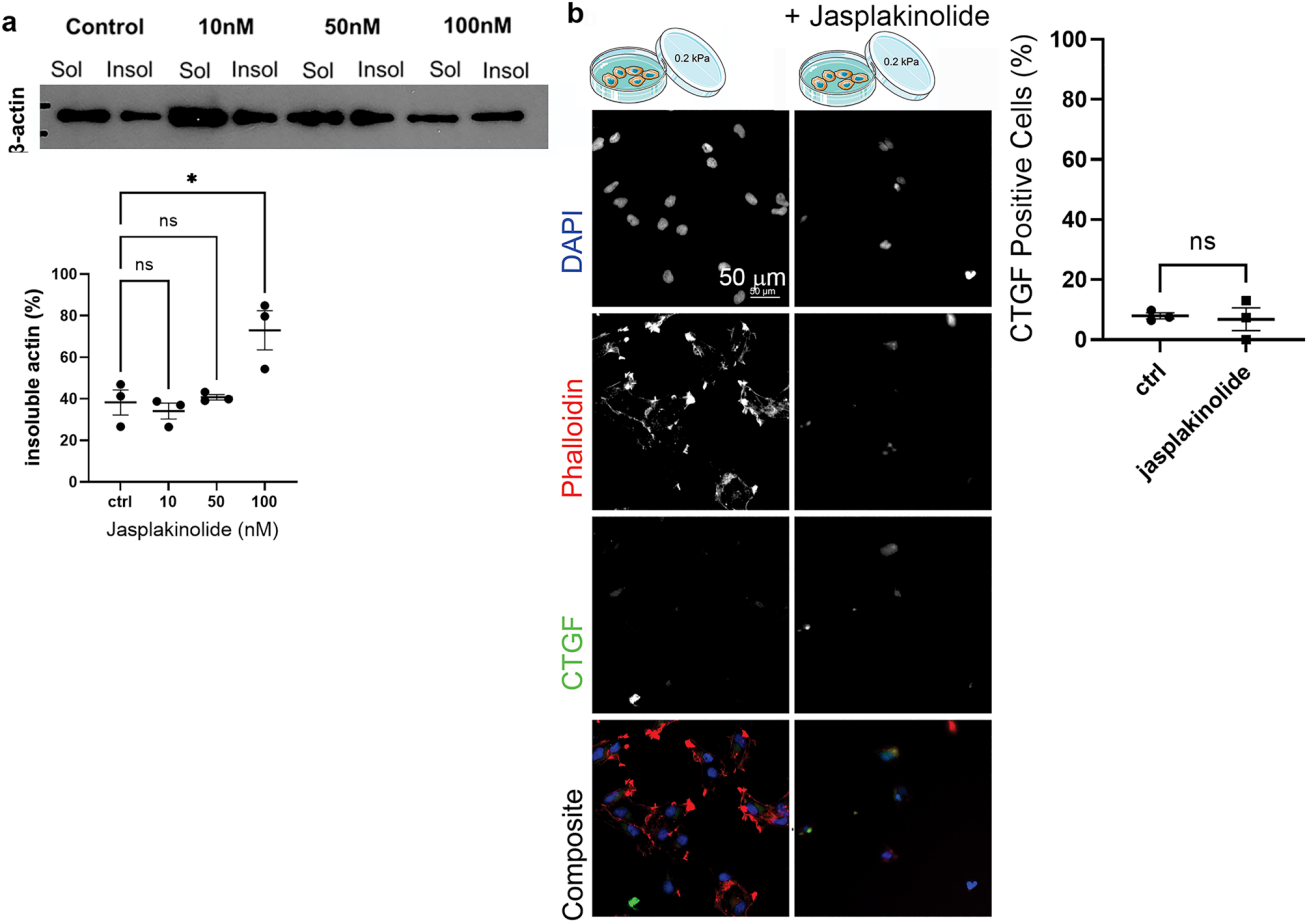
Mechanosensitive CTGF expression is independent of actin organization. (**a**) The concentration of Jasplakinolide required to stabilise actin filaments was determined by western blot analysis of the insoluble versus soluble β-actin fraction following incubation with the indicated concentrations of Jasplakinolide in cells grown on tissue culture plastic. Data points on the graph represent the insoluble actin as a percentage of total actin, collated from 3 biological repetitions. Error bars indicate ± SEM. Symbols denote statistical significance: *p < 0.05; NS = not significant (p < 0.05); One-way ANOVA with Tukey’s post-comparison test. (**b**) WK1 cells cultured on 0.2 kPa PAGs and grown in the presence of vehicle (first column of images) or 100 nM Jasplakinolide (second column of images) for 1 h, as per the schematic. Cells were fixed and immunostained with anti-CTGF, DAPI to detect cell nuclei and phalloidin to detect F-actin—shown individually in grey scale and as a colour overlay in the final row. Data points on the graph show the percentage of CTGF positive WK1 cells after an hour of culture on 0.2 kPa PAGs in the presence of vehicle (control) or 100 nM Jasplakinolide from 3 independent replicates. Error bars indicate ± SEM. Symbols denote statistical significance: N = not significant (p < 0.05); unpaired T-test.

We next investigated the reverse scenario to Lat A treatment by instead inducing actin stabilisation on soft gels with Jasplakinolide, an actin-binding cyclic peptide that increases actin nucleation^37^. Optimisation of Jasplakinolide concentration in cells grown on tissue culture plastic, demonstrated that 100 nM Jasplakinolide stabilises actin in WK1 cells (Fig. 5a). Subsequently, cells were plated and incubated overnight on 0.2 kPa gels, then treated with 100 nM Jasplakinolide or vehicle for 1 h. This approach revealed that actin stabilisation with Jasplakinolide did not induce CTGF expression in WK1 cells on soft 0.2 kPa PAGs (Fig. 5b). These data suggest that actin filament stabilisation in WK1 cells on soft substrates does not stimulate CTGF expression. Thus, mechanosensitive CTGF expression in WK1 cells does not correlate with polymerisation of the actin cytoskeleton.

## Discussion

Data in this study have established that expression of the mesenchymal marker CTGF is regulated by substrate stiffness. Although it is widely suggested that CTGF expression occurs downstream of actin polymerisation, we show that CTGF expression occurs prior to spreading and in the presence of actin depolymerising drugs and, further, is not induced by actin polymerising agents. Our study reveals that mechanosensitive CTGF proceeds independently of actin polymerisation in the GBM cells under investigation. Instead, CTGF may be a crucial mediator for F-actin formation likely via the mechanoregulated pathways that precede it, such as YAP/TAZ^30,36,38^. Accordingly, some studies report CTGF as a critical promoting factor for cell motility via F-actin stabilisation, large focal adhesion formation and lamellipodia/filopodia induction^39,40^.

The exact mechanisms underlying CTGF regulation by cytoskeletal and mechanotransduction pathways remains elusive. However, published studies suggest that RhoA seems to play an integral role^41,42^. Studies support the induction of YAP/TAZ by mechanotransduction molecules such as RhoA, independent of their canonical role in Hippo signalling pathways^26,30,36^, however, the exact mechanism of this has yet to be revealed. Neither microtubule disassembly, nor Rho kinase inhibition altered CTGF expression in WK1 cells on stiff environments (Supplementary Fig. 3), thus the causative signalling pathway still requires elucidation. Furthermore, interactions between Rho and Hippo family proteins in YAP/TAZ signalling are crucial future research targets. This is of particular interest as aberrant expression of YAP/TAZ is associated with the invasive mesenchymal type in GBM^30,36,43,44^.

While the focus of the present study has been on cellular response to the elastic qualities of the underlying PAGs, most natural ECMS and tissues have both elastic and viscous qualities. Previous studies have suggested that the morphology of LN229 primary patient-derived GBM cells differ when exposed to purely elastic versus viscoelastic matrices^45^. Magnetic Resonance Elastography measurements of GBM in vivo suggest that GBM are less viscous than the surrounding tissue. This has led to the intriguing proposition that GBM invade via viscous fingering, where a more viscous fluid is displaced by a less viscous fluid, potentially explaining the highly invasive phenotype that typifies GBM^15^. Beyond these material properties of tissue, the biochemical composition is also key. The present study has been restricted to analysis of cells cultured on Matrigel-coupled PAGs, chosen because the primary patient-derived cell lines used in this study are cultured and maintained in Matrigel-coated vessels. However, studies suggest that Hyaluronic Acid (HA)—a major component of the brain extracellular matrix—can significantly influence GBM malignancy^46^. It has been reported that the presence of HA in a soft environment can phenocopy the effects of stiffness on GBM biology^47^. Thus, in future it will be important to understand how the intersection of elasticity, viscosity and biochemical composition of the matrix contribute more broadly to GBM cell biology.

Ultimately, it could be inferred that the mechanosenstive pathways that drive CTGF expression in GBM may be dependent on upstream RhoA-mediated YAP/TAZ activity. It is important to note, however, that there was not a consistent linear correlation between YAP/TAZ nuclear localisation and CTGF expression between 8 and 50 kPa surfaces, thus the elucidation of this pathway requires further analysis. Given the mechanosensitive status of CTGF, mechanical stimuli may be capable of suppressing deregulated or aberrant signalling initiated by other activation pathways. These findings are novel in the context of GBM research and highlight the importance of mechanoregulation in cancer malignancy, as well as the need for continued research and investigation into the regulatory role of the extracellular environment in malignant cell behaviour. Not only is the stiffness of GBM not yet unequivocally established, there is also a lack of understanding of the entire range of tissue forces encountered by GBM cells as they invade the brain^48^, for example invasion along blood vessels^49^. Potentially, microregional mechanical heterogeneities in the brain may alter cellular transcription programs, pushing cells into more aggressive mesenchymal phenotypes. It may therefore be prudent to consider the entire biomechanical life cycle of GBM as they encounter microregional mechanical heterogeneities in the brain or when treated with anti-cancer therapies.

## Materials and methods

### Cell lines

The patient-derived GBM cell lines JK2, SJH1, RN1 and WK1 were kindly provided by the Brain Cancer Research Unit, QIMR Berghofer Medical Research Institute, Brisbane, Australia, from patient-derived surgical aspirate^34,50^. Patient tissue was collected by the team at QIMR following written informed consent and with human ethics approval from the Royal Brisbane and Women’s Hospital, Brisbane and the QIMR Berghofer Institute and in accordance with approved guidelines. All lines were independently Short Tandem Repeat (STR) profiled by CellBank Australia by our lab.

### Cell culture maintenance and media

All cell lines were cultured as mono-layers and maintained in filter-capped tissue culture flasks coated with 1% (v/v) Matrigel (Matrigel GFR Basement Membrane Matrix LDEV-Free; BD Biosciences, Mississauga, Canada) in Dulbecco’s Modified Eagle Medium (DMEM; ThermoFisher, MA USA). Culture vessels were coated with Matrigel by covering the internal surface with 1% Matrigel in DMEM High Glucose Pyruvate media and incubation for 1 h at 37 °C or overnight at 4 °C. Cells were maintained at 37 °C and 5% CO_2_ in KnockOut™ DMEM/F-12 (ThermoFisher) supplemented with recombinant Human EGF (20 ng/mL; ThermoFisher), recombinant Human FGFb (10 ng/mL; ThermoFisher), glutamine (20 mM/mL; ThermoFisher), penicillin/streptomyocin (100 U/mL; ThermoFisher), StemPro Neural Supplement (20 ng/mL; ThermoFisher) and heparin (20 ng/mL; Sigma-Aldrich, MO USA). Cells were sub-cultured for a maximum of 35 passages, with cells detached for passaging with StemPro Accutase (ThermoFisher).

### Polyacrylamide (PA) hydrogels

PA 35 mm Petrisoft™ (plastic-bottomed) and Softview™ (glass-bottomed) products (Matrigen, CA USA) of defined stiffness (0.5, 1, 8 and 50 kPa) were used for bright field microscopy and confocal microscopy, respectively. Stiffness calibrations are performed by Matrigen on each batch of solution of a targeted stiffness for quality assurance. All PAGs were coated with 1% Matrigel solution for 1 h and rinsed with phosphate buffered saline (PBS, ThermoFisher) before cells were seeded.

### Immunofluorescence

Cells were seeded onto Matrigel-coated PAGs and glass coverslips at a density of 7–9 × 10^6^ in supplemented media. Media was aspirated and adherent cells were washed with 1 × PBS, fixed with 4% paraformaldehyde (PFA, Sigma-Aldrich) (v/v) for 10 min at room temperature, then washed three times with 1 × PBS and permeabilised for 5 min in permeabilising solution—0.2% Triton X-100 (v/v) (Sigma-Aldrich) in wash buffer (0.5% bovine serum albumin (BSA, ThermoFisher, (w/v) in PBS).Permeabilising solution was aspirated, and the cells were washed three times with wash buffer. For TAZ staining, samples were first blocked for 1 h at room temperature with 5% BSA in normal serum (10% Donkey serum (sigma-Aldrich) (v/v) in 1 × PBS). Cells were then incubated with a primary antibody targeting CTGF (1:1000, Ab6692, Abcam) or TAZ (1:200; Ab110239; Abcam) for 1 h at room temperature followed by washing three times with wash buffer and incubation with fluorescently-tagged secondary antibody (Abcam) diluted at 1:1000 in normal serum for 1 h at room temperature. Cells were counter-stained with DAPI (Sigma-Aldrich) and fluorescently-tagged phalloidin (ThermoFisher) using standard protocols and washed a final three times with wash buffer and twice with distilled water before mounting onto microscope slides.

### Replating

For experiments involving replating, 3.2 × 10^5^ cells were seeded onto 0.2 kPa PA 35 mm plates and incubated overnight. The following day, cells were detached and replated onto fresh PAs as indicated. Fluorescent images were captured using an IX81 inverted microscope (Olympus) with an ORCA-AG ERG cooled CCD camera (Hamamatsu Photonics) at 20× magnification.

### Actin polymerisation and depolymerisation

Cells were fractionated into detergent-soluble and detergent-insoluble components as previously described^51,52^. Briefly, Soluble and insoluble fractions were isolated following detergent extraction with CSK (cytoskeleton stabilization buffer: 0.3 M sucrose, 0.5% Triton X-100, 10 mM PIPES, pH 6.8, 100 mM KCl, 1 mM CaCl_2_, 2.5 mM MgCl_2_, 1% aprotinin, 50 mM NaF, 0.1 mM Na_3_VO_40_) and 0.1% SDS-RIPA (50 mM Tris–Cl, pH 7.4, 150 mM NaCl, 5 mM EDTA, 1% Nonidet P-40, 0.1% SDS, 1% sodium deoxycholate) respectively, as previously described. Soluble and insoluble volumes were equalized and equal volumes of each loaded onto SDS-PAGE gels and separated by electrophoresis. Percentage insoluble actin is determined from densitometric measurement of insoluble actin/(soluble actin + insoluble actin). Latrunculin A (Cayman Chemical, MI USA) was prepared as a solution in Phosphate Buffered Saline (PBS) and control cells treated with matched PBS vehicle control. Jasplakinolide (Cayman Chemical, MI USA) was prepared as a solution in ethanol, and control cells treated with matched ethanol vehicle control. Separated proteins were electroblotted to PVDF membranes and membranes probed and developed with Western Lightening chemiluminescence reagent (PerkinElmer). To determine the effect of Latrunculin A treatment on mechanosensitive CTGF expression cells were first plated on 0.2 kPa gels, detached with Acutase and then plated onto 50 kPa gels in the presence of 0.5 µM Latrunculin A for 1 h. To determine the effect of Jasplakinolide, cells grown on 0.2 kPa gels were exposed to 100 nM Jasplakinolide for 1 h.

### Imaging, image processing, measurement and statistical analysis

Cells images were captured using an IX81 inverted microscope (Olympus) with ORCA-AG ERG cooled CCD camera (Hamamatsu Photonics). CTGF fluorescence intensity was analysed in ImageJ with a custom-designed analysis plugin, ‘imagej-interactive-thresholding’ (https://github.com/christophmark/imagej-interactive-thresholding), and with Metamorph image analysis software (Molecular Devices, CA USA). Cell areas were masked using the extent of their stained actin cytoskeletons, then the average fluorescence intensity of CTGF signal within these segmented areas was calculated (Supplementary Fig. 4). Background signal was calculated using negative control slides exposed to secondary antibody only, and subtracted from all slides. The data were normalised to cell number in each region of interest via concurrent nuclei counts. Statistical analysis was undertaken using Prism 8 (Graphpad). Nuclear localisation of YAP/TAZ immunostaining was scored by 3 independent, blinded scorers, assessing nuclear localisation of cells co-stained with DAPI to detect nuclei. Blinding was achieved using Blinder software^53^. Cells that were negative for YAP/TAZ immunostaining were excluded from the analysis.

## Supplementary Information

Supplementary Figures.

Supplementary Information.

## Data Availability

The datasets generated during and/or analysed during the current study are available from the corresponding author on reasonable request.
