# Supplementary material for: Mechanosensitive expression of the mesenchymal subtype marker connective tissue growth factor in glioblastoma
Source: Sci Rep. 2022 Sep 2;12:14982. doi: 10.1038/s41598-022-19175-8 (PMC9440209; doi:10.1038/s41598-022-19175-8)
Supplement: Supplementary file 1 — Supplementary Figures. [file 41598_2022_19175_MOESM1_ESM.pdf]

Mechanosensitive expression of the mesenchymal subtype marker Connective Tissue Growth Factor in Glioblastoma

Thomas James Grundy, Louise Orcheston-Findlay, Eshana de Silva, Thuvarahan Jegathees, Victoria Prior, Farhana Amy Sarker and Geraldine O'Neill

Supplementary Figures 1 - 4

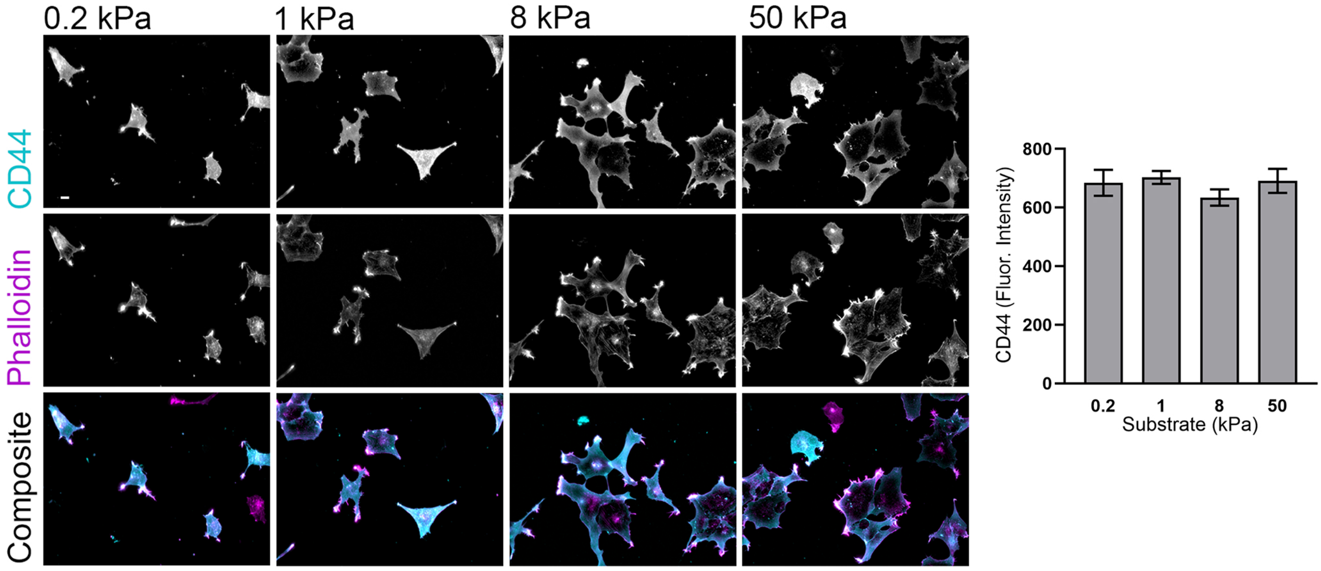

Supplementary Figure 1. CD44 expression is not mechanosensitive. WK1 cells seeded onto matrix-coupled polyacrylamide (PA) hydrogels (PAGs) of varying stiffness (0.2, 1, 8 or 50 kPa) and immunostained for CD44. Scale bar 20 µm. Histogram shows the mean fluorescent intensity of CD44 immunostaining expressed relative to the cell area. One-way ANOVA = not significant. Error bars indicate SEM.

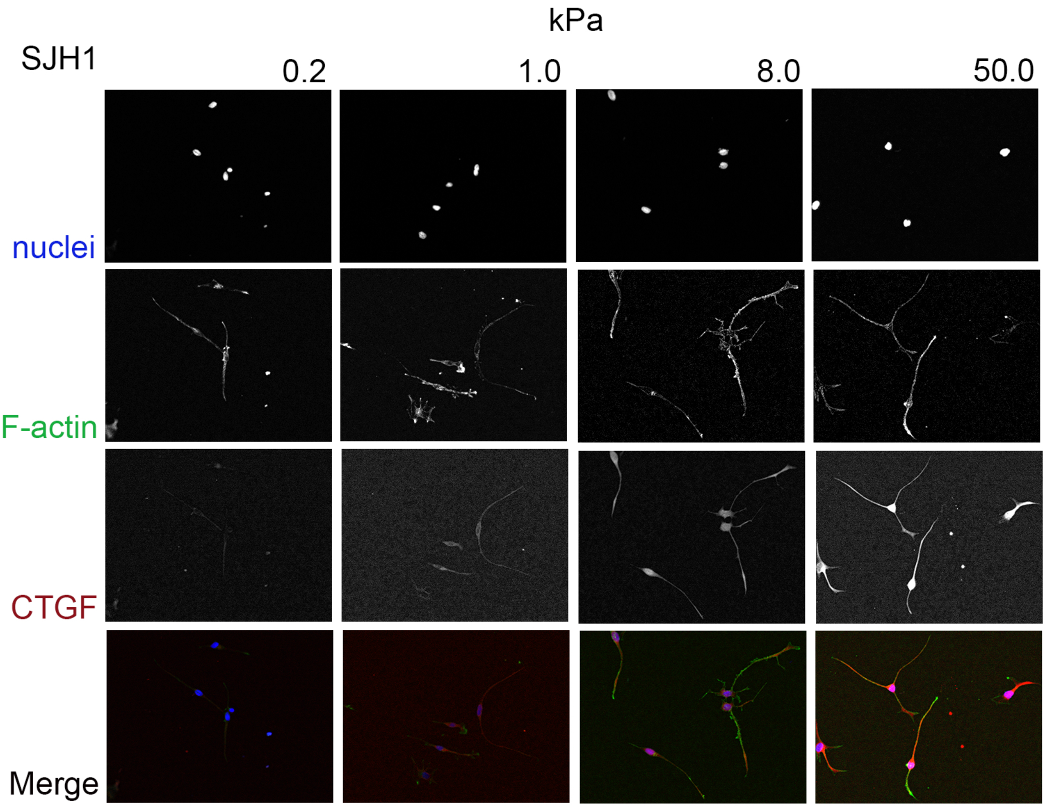

Supplementary Figure 2. Mechanosensitive expression of CTGF in SJH1 cells. Images show SJH1 cells plated on the indicated PAGs and immunostained with DAPI to detect nuclei, fluorescently-tagged phalloidin to detect filamentous (F-actin) and CTGF.

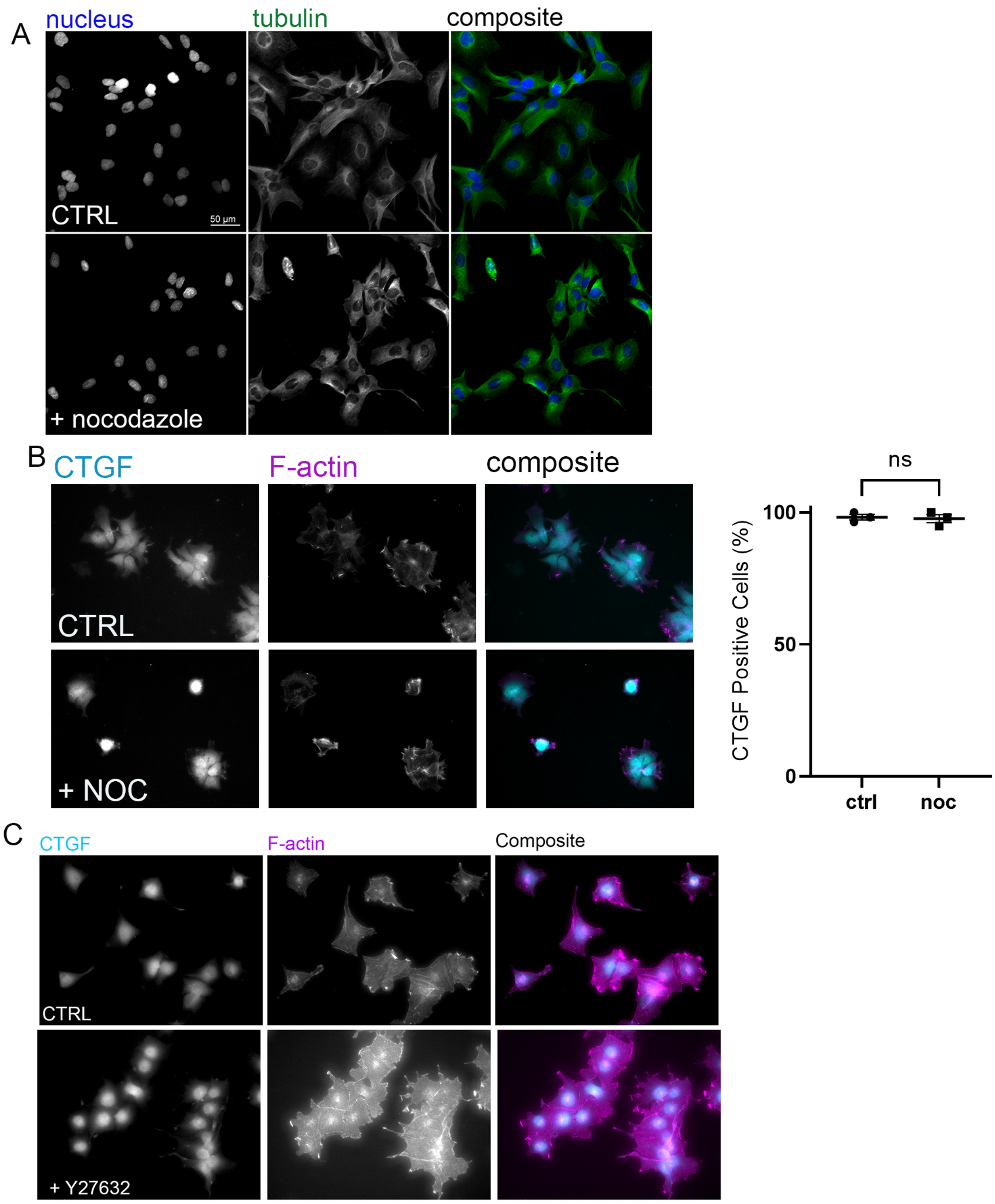

Supplementary Figure 3. Neither microtubule disruption nor Rho kinase inhibition change CTGF expression. A. Images showing that exposure of WK1 cells plated on glass coverslips for 1 hour to 1 µm nocodazole treatment causes microtubule disassembly. Cells stained with DAPI to detect nuclei and anti-polymerized tubulin antibodies to reveal the microtubule network. B. WK1 cells were grown overnight on 0.2kPa PAGs, detached and replated onto 50kPa gels in the presence of media containing 1 µm nocodazole for 2 hours, followed by immunostaining for CTGF and F-actin expression. Data points on graph show the percentage of CTGF positive cells in 3 independent experiments. NS = not significant, Students' t-test. C. WK1 cells grown on glass coverslips were treated with 10 µm Y-27632 for 1 hour to inhibit ROCK. Cells immunostained with antibodies to CTGF and phalloidin to detect F-actin as indicated.

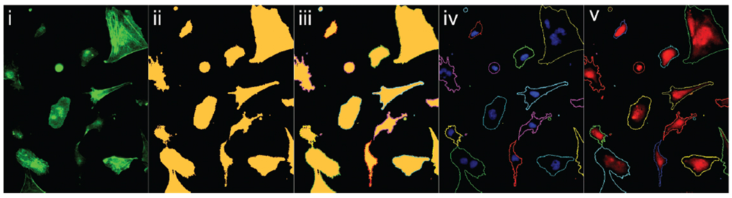

Supplementary Figure 4. Average-intensity analysis of immunofluorescent staining. (i) Cells counter-stained with phalloidin, which binds filamentous actin. (ii) Cell segmentation based upon phalloidin counterstain. (iii) Detection and outlining of segmented regions. Only cell-sized regions were analysed further. (iv) Cell nuclei (counterstained with DAPI) relative to cell regions. (v). Protein of interest, relative to cell regions.
