# Supplementary figures and images for: Mechanosensitive expression of the mesenchymal subtype marker connective tissue growth factor in glioblastoma
Source: Sci Rep. 2022 Sep 2;12:14982. doi: 10.1038/s41598-022-19175-8 (PMC9440209; doi:10.1038/s41598-022-19175-8)

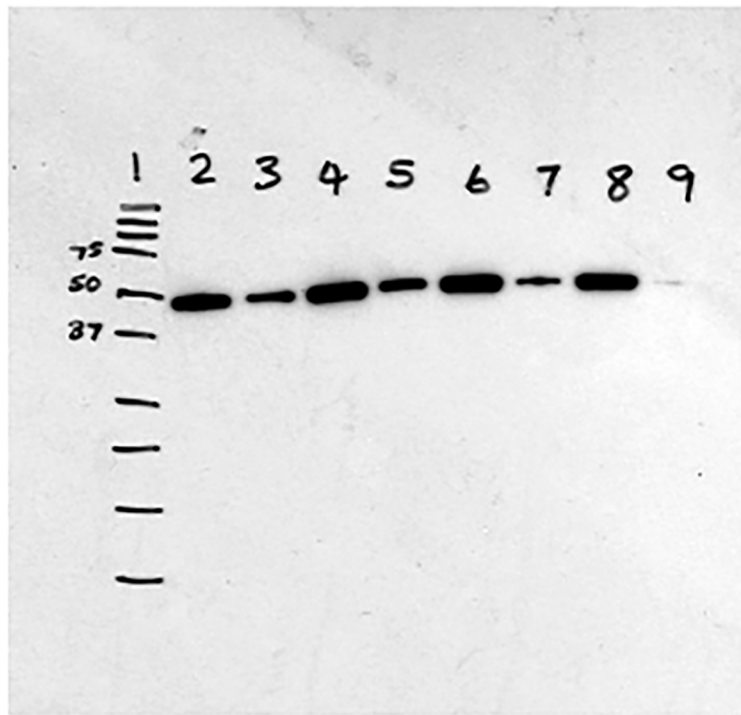

Uncropped western blot from Figure 4A

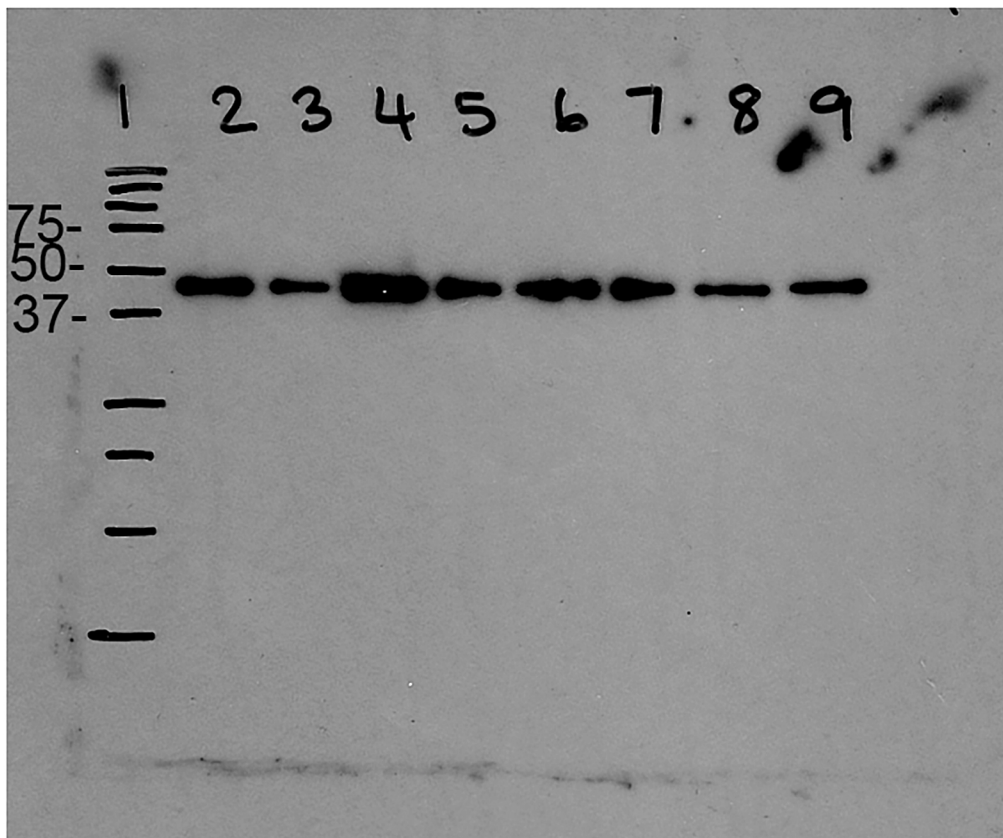

Uncropped western blot from Figure 5A

Supplement: Supplementary file 2 — Supplementary Information. [file 41598_2022_19175_MOESM2_ESM.pdf]
